# Liquefaction of *Ruscus aculeatus* Branches into Bio-Polyols: Process Optimization and Polyol Characterization

**DOI:** 10.3390/polym18070880

**Published:** 2026-04-03

**Authors:** Yuliya Dulyanska, Luísa Cruz-Lopes, Fábio Bernardo, Dmitry V. Evtuguin, Raquel P. F. Guiné, Fernando J. Gonçalves, Luís A. E. Batista de Carvalho, Maria João Barroca, Bruno Esteves

**Affiliations:** 1 CERNAS—IPV Research Centre and Agrarian School, Polytechnic University of Viseu, Campus Politécnico, Repeses, 3504-510 Viseu, Portugal; ydulyanska@esav.ipv.pt (Y.D.); raquelguine@esav.ipv.pt (R.P.F.G.); fgoncalves@esav.ipv.pt (F.J.G.); 2 Molecular Physical-Chemistry, LAQV—REQUIMTE, Department of Chemistry, University of Coimbra, 3004-535 Coimbra, Portugal; labc@ci.uc.pt (L.A.E.B.d.C.); mjbarroca@esac.pt (M.J.B.); 3 CERNAS—IPV Research Centre and ESTGV—Viseu School of Technology and Management, Polytechnic University of Viseu, Campus Politécnico, Repeses, 3504-510 Viseu, Portugal; bruno@estgv.ipv.pt; 4 CICECO—Aveiro Institute of Materials and Department of Chemistry, University of Aveiro, 3810-193 Aveiro, Portugal; fabiobernardo@ua.pt (F.B.); dmitrye@ua.pt (D.V.E.); 5 ESAC—Coimbra Agricultural School, IPC—Polythecnic University of Coimbra, Bencanta, 3045-601 Coimbra, Portugal

**Keywords:** bio-polyols, hydroxyl number, liquefaction, *Ruscus aculeatus* L., thermal analysis, viscosity

## Abstract

The conversion of lignocellulosic biomass into bio-polyols through liquefaction has attracted increasing interest as a sustainable route for polymer feedstock production. The liquefaction of *Ruscus aculeatus* L. branches was investigated to identify optimal processing conditions and to evaluate the properties of the resulting bio-polyols. The effects of temperature, reaction time, particle size, and material-to-solvent ratio on liquefaction yield were systematically studied. Liquefaction yield increased markedly with temperature, reaching up to 92% at 180 °C after 60 min of reaction, while reaction time showed only a marginal effect beyond 15 min. Smaller particle sizes and higher solvent ratios improved liquefaction efficiency, with optimal conditions identified between 1:7 and 1:10 material-to-solvent ratios. The hydroxyl number decreases with increasing liquefaction temperature due to dehydration and condensation reactions. Thermal and rheological analyses indicated improved thermal stability and increased viscosity at higher liquefaction temperatures. These results highlight the potential of *Ruscus aculeatus* branches as a promising renewable feedstock for bio-polyol production and polyurethane applications.

## 1. Introduction

Driven by the growing demand for sustainable materials, the development of bio-based polymers has attracted significant scientific interest. In this context, *Ruscus aculeatus* L. (Butcher’s Broom), a native Portuguese plant traditionally used in medicine, represents a potentially valuable lignocellulosic resource for biomass valorization [1]. While previous studies have mainly focused on its extractive compounds, the valorization of its structural macromolecular components has not yet been extensively explored.

Polyalcohol liquefaction is a thermochemical process used to convert solid biomass into liquid products by reacting with polyhydric alcohols such as glycerol, ethylene glycol, or propylene glycol [2,3,4]. This approach has gained increasing attention as an efficient route for converting biomass residues into valuable intermediates [5,6,7,8]. During liquefaction, the solvent acts both as a reaction medium and a reactive agent, promoting the depolymerization of lignocellulosic structures into lower-molecular-weight compounds. The resulting bio-polyols can be used for producing resins, adhesives, polyurethane foams, and other polymeric materials [9,10,11].

The hydroxyl number (OH-index) is a key parameter in polyol characterization, particularly for polyurethane (PU) production, as it influences the crosslinking density, mechanical properties, and reactivity of the resulting polymers. Higher OH indices generally lead to more rigid polyurethanes, whereas lower values promote more flexible materials. For instance, Lim et al. [12] reported that rigid polyurethane foams formulated with polyols of increasing hydroxyl number exhibit enhanced compression strength and dimensional stability, which was stated to be due to a higher crosslink density arising from more allophanate linkages. Therefore, controlling the OH index of polyols is essential to tailor PU properties for specific applications, from rigid foams and coatings to flexible elastomers. Additionally, higher OH indices lead to a decrease in the average cell size, and the closed-cell content rises, which further reinforces the material [12].

The OH index of liquefied polyols has been proven to depend both on the original biomass and on the type and amount of polyalcohols used as solvents. For example, the liquefaction of eucalypt and pine woods leads to different hydroxyl numbers of 304 mg KOH/g for pine and 277 mg KOH/g for eucalypt, showing the different nature of the biomass [13]. Additionally, Zheng et al. [14] reported OH indices of 700 mg KOH/g for polyols from liquefied pine wood when using a polyethylene glycol–glycerol mixture, compared to 280 mg KOH/g with polyethylene glycol alone. In addition to solvent choice, the presence of impurities in the solvent can further affect the OH index of the resulting polyols [15]. OH-index has been seen to increase for higher particle sizes [16]. Studies with walnut shells showed that the lowest OH-index was observed for the smaller particles of 0.3–0.6 mm.

Polyurethane foam formation and final properties are strongly influenced by the initial viscosity of the polyol component, which governs early-stage bubble nucleation, cell growth, and drainage before significant polymer network development occurs. Variations in initial polyol viscosity directly affect foam morphology, density, mechanical performance, and dimensional stability by setting the viscoelastic resistance of the reacting system at the onset of foaming [17,18,19]. During the early foaming stage, gas generation and cell expansion occur while the system viscosity is still dominated by the polyol phase. A higher initial polyol viscosity limits bubble growth, suppresses drainage, and reduces cell coalescence, leading to finer and more uniform cellular structures [17]. In contrast, low-initial-viscosity polyols allow for rapid cell expansion and prolonged liquid-phase mobility, increasing the likelihood of cell coarsening and collapse prior to gelation [17].

Higher-viscosity polyols have been associated with changes in foam apparent density and viscosity-dependent compressive strength responses, including cases of density reduction and property recovery at higher substitution levels [18,20]. While increased initial viscosity can improve dimensional stability, excessively high viscosities may hinder mixing and handling, motivating industrial formulation strategies that balance initial viscosity through polyol blending or the addition of low-molecular-weight components [19,21].

Particle size is an important parameter influencing biomass liquefaction efficiency. Smaller particles generally provide a larger specific surface area, which enhances solvent penetration and improves mass transfer between the liquefaction medium and the lignocellulosic matrix [22]. This facilitates the acid-catalyzed cleavage of glycosidic bonds in cellulose and hemicellulose as well as ether linkages in lignin, promoting more efficient depolymerization. However, particle size is not the only determining factor, as variations in chemical composition across different particle size fractions can also influence the overall liquefaction performance, as seen before [11,23].

The objective of this work is to contribute to the eco-valorization of *Ruscus aculeatus* L. by thoroughly investigating and optimizing the liquefaction process of its branches to produce bio-based polyols, and by evaluating the suitability of these polyols as precursors for polyurethane foams. While biomass liquefaction for polyol production is widely studied, the novelty of this work lies in the exploration of *Ruscus aculeatus* as an unconventional and underutilized lignocellulosic resource. This species is abundant in Mediterranean regions and generates pruning residues that are currently unexploited, representing a low-cost and locally available feedstock. By demonstrating its potential as a viable raw material for polyurethane applications, this study contributes to expanding the range of alternative biomass sources and supports the development of more region-specific and sustainable bio-based value chains.

## 2. Materials and Methods

### 2.1. Materials

#### Samples

Samples of *Ruscus aculeatus* L. branches were harvested at ripening time in December 2022. The samples were milled in a Retsch SMI mill (Retsch GmbH, Haan, Germany) and sieved using a Retsch AS200 (Retsch GmbH, Haan, Germany) for 20 min at 50 rpm. Four fractions, >40 mesh (420 µm), 40–60 mesh (250–420 µm), 60–80 mesh (177–250 µm), and <80 mesh (177 µm), were obtained.

### 2.2. Liquefaction

Liquefaction experiments were conducted using a jacketed batch reactor Parr 5100, with a total volume of 600 mL supplied by Parr Instruments Co. (Moline, IL, USA). For each run, 10 g of oven-dried material, previously milled to a particle size range of 0.250–0.420 mm, was loaded into the reactor. To assess the influence of particle size on liquefaction efficiency, additional experiments were carried out using biomass fractions classified as >40, 40–60, 60–80, and <80 mesh. Finer particle sizes typically enhance liquefaction efficiency by increasing surface area and improving mass transfer. Prior to heating, the reaction mixture was thoroughly homogenized, and mechanical agitation was applied at a constant stirring speed of 75 rpm throughout the process. The liquefaction medium consisted of a 50:50 (*w*/*w*) mixture of glycerol and ethylene glycol, selected for their economic viability and comparatively low environmental footprint. Sulfuric acid was added as a catalyst at a concentration corresponding to 3 wt% of the total solvent mass.

Reaction temperature was controlled by thermal oil circulating through the reactor jacket. The effective liquefaction time was defined as the period after the system reached the target temperature (140 °C, 160 °C, or 180 °C). Reaction times ranged from 15 to 60 min, depending on the experimental conditions investigated. Upon completion, the reactor was rapidly cooled using an ice bath to quench the reaction.

The selected temperature range (140–180 °C) was based on operational conditions commonly reported for acid-catalyzed liquefaction of lignocellulosic biomass. Within this interval, depolymerization reactions proceed efficiently while avoiding excessive thermal degradation and char formation that may occur at higher temperatures. The resulting liquefied products were diluted with 100 mL of methanol and subsequently filtered under vacuum using a Büchner funnel fitted with filter paper (pore size 0.6 µm). The solid residue was further rinsed with approximately 200 mL of distilled water to eliminate residual solvent. Finally, the insoluble fraction was dried and quantified gravimetrically to determine the liquefaction yield.

All liquefaction experiments were performed in duplicate. Standard deviations were calculated and are presented as error bars in the figures to indicate experimental variability and ensure the statistical reliability of the measurements.

### 2.3. Determination of Hydroxyl Number

The hydroxyl number (IOH) of the liquefied material was quantified following an acetylation–titration procedure adapted from previously reported methodologies [24]. The method is based on the esterification of free hydroxyl groups and the subsequent potentiometric titration of the unreacted acetic acid. Briefly, approximately 20 mg of liquefied material was weighed into glass test tubes, to which 0.1 mL of an acetylating reagent was added. The acetylating mixture was freshly prepared by combining acetic anhydride (2.35 mL) with pyridine (2.00 mL). The reaction mixtures were thoroughly homogenized and incubated in a temperature-controlled oven at 50 ± 2 °C for 24 h to ensure complete acetylation of the hydroxyl groups. After the reaction period, the samples were allowed to cool to room temperature, followed by the addition of 10 mL of acetone and 10 mL of distilled water to quench the reaction and remove excess reagents. The resulting solution was then titrated with a standardized 0.1 N lithium hydroxide (LiOH) solution using potentiometric detection. A blank containing only the acetylating mixture was analyzed under identical conditions.

The hydroxyl content was calculated using Equations (1) and (2):

(1) $$OH\left(\%\right)=\frac{\left[\left(ms\times \frac{Vb}{mb}\right)-V\right]\times f\;\times 1.7\times 100}{W}$$

(2) IOH(mg KOH/g)=33×OH(%)

where ms is the mass of the acetylating mixture (mg), Vb is the volume of LiOH solution used for the blank titration (mL), mb is the mass of the blank (mg), V is the volume of LiOH solution consumed during sample titration (mL), W is the mass of the sample (mg), f is the correction factor for the standardized LiOH solution, and 1.7 mg corresponds to the mass of hydroxyl groups equivalent to 1 mL of 0.1 M LiOH. The constant 33 was applied to convert the hydroxyl percentage into the hydroxyl number (IOH) expressed as mg KOH g^−1^.

### 2.4. Thermogravimetric Analysis

Thermogravimetric analysis (TGA) and differential scanning calorimetry (DSC) were performed to investigate the thermal stability, mass loss behavior, and thermal transitions of the samples. The measurements were carried out using a PerkinElmer STA 6000 Simultaneous Thermal Analyser (PerkinElmer, CT, USA). An initial sample mass of approximately 10 mg was placed in a platinum crucible and heated from 20 °C to 600 °C under a nitrogen atmosphere with a flow rate of 20 mL/min to prevent oxidative degradation. The mass change (TGA) and heat flow (DSC) were continuously recorded as functions of temperature. At the end of the experiment, a residual mass of approximately 4 mg was obtained.

### 2.5. Viscosity–Temperature Behavior

The viscosity–temperature behavior of the bio-polyols was evaluated using a Rapid Visco Analyzer (RVA 4500, Perten Instruments, Stockholm, Sweden), operating under controlled temperature and rotational conditions.

Although the RVA is commonly used for starch-based systems, it functions as a controlled-shear rotational viscometer that determines apparent viscosity based on torque resistance during sample flow. Therefore, it is suitable for comparative rheological profiling of viscous liquid systems such as bio-polyols, although the results represent apparent viscosity under instrument-specific shear conditions.

Prior to analysis, all samples were dried to eliminate the influence of residual moisture on viscosity measurements. A fixed mass of 20.0 ± 0.01 g of polyol was introduced into the aluminum canister.

The measurement protocol consisted of an initial high-speed mixing step at 960 rpm for 10 s to ensure sample homogenization, followed by a constant rotational speed of 160 rpm throughout the test. Although the RVA does not provide direct shear rate values, the controlled rotational regime ensures consistent shear conditions across all experiments. Thus, the measurements should not be interpreted as absolute rheological parameters, but as reproducible comparative data obtained under controlled thermo-mechanical conditions.

The analysis was conducted using a standardized heating–cooling cycle. Initially, the sample was equilibrated at a low temperature to establish baseline viscosity. The temperature was then increased at a controlled rate to a predefined maximum, followed by a short holding period and subsequent cooling back to the initial temperature. Apparent viscosity (cP) was continuously recorded as a function of time and temperature.

All measurements were performed in triplicate to ensure reproducibility. The results are presented as comparative viscosity profiles, enabling consistent evaluation of the influence of liquefaction conditions on polyol behavior

### 2.6. FTIR-ATR

The material obtained from the liquefaction of *Ruscus aculeatus* L. branches was characterized by Fourier Transform Infrared Spectroscopy equipped with Attenuated Total Reflectance accessory (FTIR-ATR). Prior to analysis, the samples were dried in an oven at 100 °C for 8 days to ensure the complete removal of residual moisture.

FTIR-ATR (Fourier transform infrared in attenuated total reflectance mode) spectra were recorded in duplicate in the mid-IR range (400–4000 cm^−1^) using a Bruker Optics Vertex 70 FTIR spectrometer, equipped with a Bruker Platinum ATR single-reflection diamond accessory, and purged by CO_2_-free dry air. A Ge on KBr substrate beamsplitter and a liquid nitrogen-cooled wide-band mercury cadmium telluride (MCT) detector were employed. Each spectrum was obtained by averaging 128 scans at a spectral resolution of 2 cm^−1^, using the three-term Blackman–Harris apodization function. Spectral acquisition and processing were performed using Bruker OPUS software (version 8.1), which was also used to remove residual H_2_O and CO_2_ contributions and correct the spectra regarding the wavelength dependence of the penetration depth of the electric field in ATR (for a mean refractive index of 1.25). The spectra for the liquefied material were normalized at 1045 cm^−1^.

## 3. Results and Discussion

### 3.1. Liquefaction

In order to study the ideal parameters for the liquefaction of *R. aculeatus* L. branches, different conditions were tested. The results evidenced demonstrate a clear increase in the liquefaction yield as temperature rises: from 39% at 140 °C to 67% at 160 °C, and to 92% at 180 °C. The data not just confirms that rising temperature significantly increases the liquefaction yield as it also shows that this effect is slightly more expressive between 140 °C and 160 °C, in which the liquefaction yield improves by +28% as compared to its increase of +25% between 160 °C and 180 °C. Elevated temperatures promote protonation and cleavage of glycosidic linkages in cellulose and hemicellulose, as well as ether bond scission in lignin structures, facilitating biomass solubilization and increasing conversion efficiency. In contrast to *R. aculeatus* branches, which show a steep, temperature-driven rise in liquefaction yield with the temperature increase, olive branches studied in equivalent conditions [25] display a more moderate response, from 52.5% to 80.9% across the same temperature range. Likewise, the liquefaction or *Arbutus unedo* bark leads to significantly lower liquefaction percentages at similar conditions [26]. On the other hand, the liquefaction of *Cytisus scoparius* obtained a slightly higher percentage (95%) [27]. These differences can be attributed to different chemical compositions since, as reported before by Hu et al. [28], hemicelluloses, lignin, and amorphous cellulose exhibit higher structural disorder at the molecular level, which leads to a significant enhancement of the chemicals’ mass transport towards their surface, supporting their faster degradation. On the other hand, the molecular arrangement of crystalline cellulose might hinder its degradation for a longer period, requiring prolonged reaction time.

In order to study the time effect on the liquefaction yield, another set of experiments was performed at the maximum selected temperature (180 °C). The corresponding results clearly demonstrate that increasing the reaction time leads to a slight improvement in the liquefaction yield, increasing from 89% at 15 min to 90% at 30 min, and even to 92% at 60 min. Besides that, a reaction time of only 15 min is enough to sustain the reaction process, allowing us to obtain almost 90% of liquefaction yield, which is a valuable result for the industrial application of the liquefaction process. This limited variation indicates that depolymerization reactions proceed rapidly at 180 °C, with most bond cleavage occurring within the first 15 min. The system appears to approach near-complete conversion quickly, suggesting that temperature is the dominant kinetic driver under these conditions. As a result, the remaining 45-min period exhibits a slight improvement in the liquefaction yield. Despite this tangential improvement, a remarkable optimization is attained by saving energy by subsequently reducing the reaction time to a quarter without significantly diminishing the liquefaction yield. The liquefaction percentage obtained for 15 min (180 °C) is much higher than, for example, the liquefaction of *Arbutus unedo* bark with approximately 30% [26] or olive branches with 69% [25] at similar conditions or for eucalyptus branches for 30 min at the same temperature with around 40% [29]. Reaction times between 15 and 60 min were selected based on typical liquefaction durations reported in the literature. Extending reaction time beyond this range generally provides limited additional improvement in liquefaction yield while increasing energy consumption and promoting secondary condensation reactions as reported before [30].

**Figure 1:**
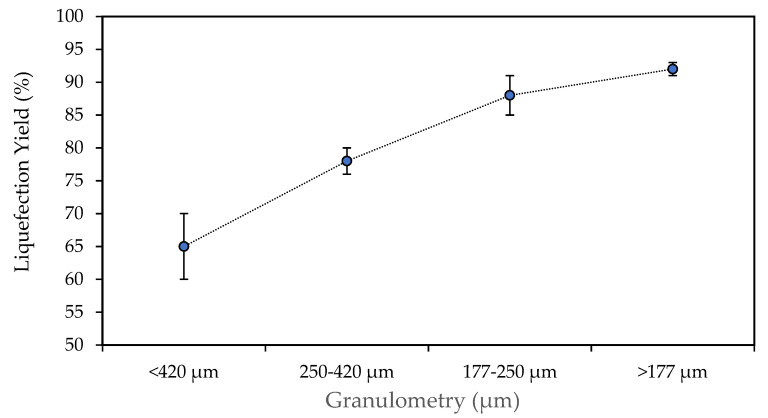
Effect of biomass particle size on the liquefaction yield of *Ruscus aculeatus* branches. Experiments were conducted at 180 °C for 60 min using a glycerol/ethylene glycol (1:1 *w*/*w*) solvent mixture with 3 wt% sulfuric acid catalyst. Error bars represent standard deviations.

Another operational parameter affecting the liquefaction yield concerns the granulometric profile of the biomass raw material inserted into the reaction at the beginning of the whole process. Thus, as it is possible to observe from Figure 1, the liquefaction yield grows as the average particle size is progressively smaller, which is probably due to the higher contact surface between the chemicals and biomass. In accordance with Moreira et al. [22], smaller particle sizes typically improve heat and mass transfer, allowing solvents to penetrate more easily and catalysts to access reactive sites more effectively, thereby enhancing biomass conversion efficiency. These results demonstrate that liquefaction is not solely governed by chemical kinetics but is significantly influenced by physical factors such as particle size and interfacial contact. In larger particles, internal regions may remain partially inaccessible, limiting complete depolymerization. As particle size decreases, diffusion resistance is reduced, allowing for more uniform acid-catalyzed cleavage throughout the biomass matrix. Besides that, smaller particles present higher specific surface area and greater exposure of amorphous regions. Similar results were presented before for the liquefaction of walnut shells with glycerol for 120 min at 150 °C [16] or for pine wood chips [31]. Nevertheless, this is not always the case since the different chemical compositions exhibited by each distinct granulometric fractions can be more important than the effect of the particle size, as reported before for cork [11,32]. These results suggest that there are no significant chemical changes in *Ruscus* branches’ chemical composition across different granulometries.

**Figure 2:**
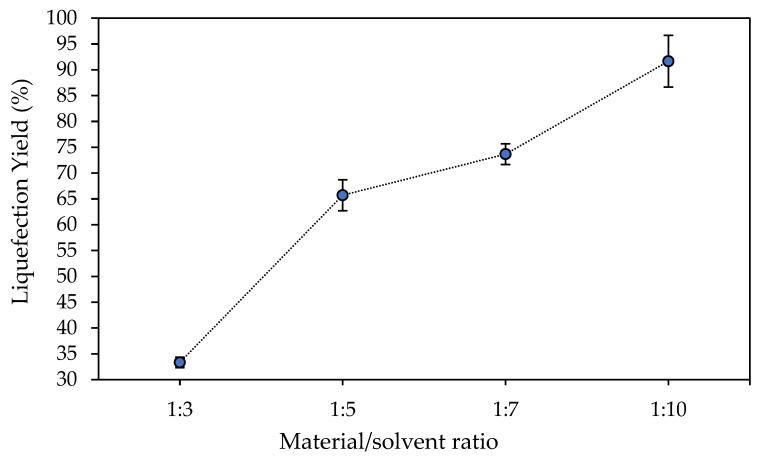
Effect of the material-to-solvent ratio on liquefaction yield of *Ruscus aculeatus* branches at 180 °C for 60 min using a glycerol/ethylene glycol (1:1 *w*/*w*) solvent mixture and 3 wt% H_2_SO_4_ catalyst. Error bars represent standard deviations.

The remaining operational parameter generally affecting the liquefaction yield refers to the material-to-solvent ratio (material:solvent). The use of lower amounts of solvent is advantageous from an industrial point of view. In order to explore its effect, a set of experiments using 1:3, 1:5, 1:7 and 1:10 ratios were carried out, and the corresponding results are highlighted in Figure 2. The results presented reveal that the liquefaction yield increases successively by raising the amount of solvent used to perform the experiment. At low solvent ratios (e.g., 1:3), the system likely becomes highly viscous early in the reaction, limiting mass transfer and diffusion of acid and solvent into biomass particles. Increasing the solvent content reduces system viscosity and enhances solvent penetration into the lignocellulosic structure, facilitating mass transfer and biomass depolymerization. From an optimization perspective, material-to-solvent ratios between 1:7 and 1:10 are commonly reported as suitable for industrial applications because they provide high liquefaction yields while maintaining reasonable solvent consumption. The 1:7 ratio was considered to be the ideal for the liquefaction of southern pine with a 70/30 mixture of PEG 400/glycerol [33]. Similar ratio of approximately 1:6 was used to liquefy Kraft lignin [34]. Thus, this behavior might be explained through a more intense solvation effect while using higher solvent ratios. The presence of higher amounts of solvent compared to the material fraction provides highest mass transfer gradients towards the solvent bulk constantly refreshing the material’s surface with solvent molecules. As this experiment was planned and executed at a temperature of 180 °C, for 60 min under intense stirring, the combined mechanical and thermal energy source might result in mass transfer intensification, by increasing the solvent’s ability to dissolve the material’s degrading products, as well as by constantly renewing the material’s surface with fresh solvent from the reaction medium. Then, from an optimization perspective, the most adequate ratio to be considered or recommended for an industrial purpose centers around material-to-solvent ratios ranging from 1:7 to 1:10.

When compared with other lignocellulosic biomass liquefaction studies reported in the literature, the liquefaction yields obtained for *Ruscus aculeatus* branches are relatively high under similar reaction conditions [25,26]. The yield of approximately 92% obtained in the present study therefore indicates that *Ruscus aculeatus* branches represent a highly suitable lignocellulosic feedstock for bio-polyol production under acid-catalyzed liquefaction conditions.

### 3.2. Hydroxyl Number

Hydroxyl Value or Hydroxyl Number represents a standard measure of the amount of hydroxyl groups (OH groups) present in a bio-polyol sample. This quantity is currently used as a method to evaluate the liquefaction performance as well as the material’s suitability to be used as a reactant for the production of polyurethane foams. The variation in hydroxyl number with the temperature of liquefaction was studied for polyols liquefied at temperatures of 140 °C, 160 °C, and 180 °C, for a constant 60-min period.

The results show that the hydroxyl number of the polyol liquefied at 140 °C is the highest with 1038 mg KOH∙g^−1^. As the temperature increases from 140 °C to 160 °C, a decrease to 932 mg KOH∙g^−1^ is observed, and there is a further decrease to 916 mg KOH∙g^−1^ for 180 °C. This trend is particularly significant because it contrasts with the increase in liquefaction yield. While higher temperatures promote more extensive depolymerization and solubilization, they simultaneously reduce the hydroxyl number per unit mass of product. Similar results were presented before for the liquefaction of several wood, namely poplar, oak, spruce and beech [30]. These authors stated that the reduction in hydroxyl (OH) number is attributed to the dehydration and thermal oxidation of glycols, as well as condensation reactions occurring between the glycols and wood components such as cellulose, hemicelluloses, and lignin. Supporting this interpretation, Yao et al. [35] subjected glycols alone to the same liquefaction conditions and measured their OH numbers, finding no significant change. This result indicates that the primary decrease in the OH number of the reaction mixture is mainly due to reactions between the glycols and the wood components present. Although temperature enhances initial bond cleavage, it also may promote secondary condensation reactions, especially among lignin-derived fragments. Similar results were presented before by Gosz et al. [36] for the liquefaction of alder wood. As these authors stated, an increase in reaction time or temperature is accompanied by a decrease in hydroxyl number and an increase in biomass conversion. These authors attributed the decrease in hydroxyl number to dehydration or condensation of the liquefaction solvents, and/or thermal oxidation reactions between the solvents and lignocellulosic biomass components.

Different results were presented by Amran et al. [37], who obtained an increase in the hydroxyl number from 177.6 to 323.2 mg KOH/g as the temperature rose from 130 °C to 160 °C, although further increasing the temperature led to a decrease in hydroxyl number. Also, these authors used a mixture of PEG and glycerol that has an initial OH number much lower than the ethylene glycol/glycerol mixture. In another study, Jin et al. [2] stated that the hydroxyl number of the liquefied enzymatic hydrolysis lignin generally decreased as the liquefaction temperature increased. The hydroxyl number dropped from 329 mg KOH/g at 130 °C to 191 mg KOH/g at 170 °C, indicating, in accordance with these authors, that higher temperatures promote recondensation reactions that reduce available hydroxyl groups. At the same time, extending the liquefaction time at 130 °C leads to a gradual decrease in hydroxyl number, from 315 mg KOH/g at 1.5 h to 224 mg KOH/g at 3 h, suggesting that extended reaction times also promote secondary reactions that reduce the hydroxyl-group content. These trends demonstrate that both higher temperatures and longer reaction times tend to lower the hydroxyl number, highlighting the importance of optimizing these parameters for maximizing polyol functionality.

In order to evaluate the time effect in the amount of hydroxyl groups obtained from the biomass material’s degradation during the liquefaction process, another set of experiments was performed at a temperature of 180 °C. Thus, liquefaction experiments were carried out for the *R. aculeatus* L. branches biomass sample for consecutive 15, 30, and 60-min periods, obtaining the OH numbers. At the liquefaction temperature of 180 °C, by increasing the reaction time from 15 min to 30 min, the OH number rises slightly from 917.97 mg KOH∙g^−1^ to 935.02 mg KOH∙g^−1^, but decreases afterwards to 915.62 mg KOH∙g^−1^ of the sample’s weight after a 60-min period of reaction. The initial rise from 15 to 30 min suggests that depolymerization and formation of hydroxyl-rich fragments predominate during the early stage of liquefaction. However, as reaction time increases further, secondary condensation and etherification reactions become more relevant, consuming hydroxyl groups and reducing the hydroxyl number. Generally, most of the studies refer to a decrease in OH value along liquefaction time; for instance, the work of Kurimoto et al. [38], where the hydroxyl numbers linearly decreased from 210 to 100 mg KOH/g with the increase in reaction time. Nevertheless, the essays are generally done for a more prolonged time, like for example the work done by Jin et al. [2], where the studied times ranged from 1 h to 6 h. In this case, at the beginning of the liquefaction, there is an equilibrium between the production of new hydroxyl groups and the consumption of the large amount of initial hydroxyl groups from liquefying solvents due to degradation during liquefaction. Similar results were presented before for the liquefaction of *Eucalyptus globulus* bark at 150 °C, where the hydroxyl number increased between 20 and 85 min, indicating the depolymerization of the biomass into the reaction medium but a decrease afterwards [39], or in the work by Lu et al. [40], where the hydroxyl number increases from 267.0 to 319.8 mg KOH/g as the liquefaction time is extended from 15 to 60 min.

**Figure 3:**
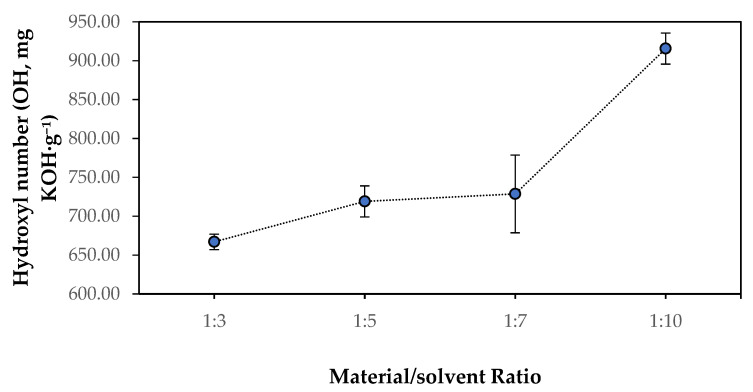
Effect of the material-to-solvent ratio on the hydroxyl number (OH, mg KOH·g^−1^) of polyols obtained from liquefaction of *Ruscus aculeatus* branches at 180 °C for 60 min. Error bars represent standard deviations.

The material-to-solvent ratio is considered an important operational parameter to study in order to establish a better understanding of the OH number evolution during the liquefaction process. Results in Figure 3 show that the OH number increases as the solvent content rises. The increase is moderate for ratios of 1:3, 1:5, and 1:7, ranging from 667 to 729 mg KOH·g^−1^. However, a substantial increase occurs between the 1:7 and 1:10 ratios, with the OH number reaching 916 mg KOH·g^−1^. This is to be expected since the high hydroxyl number of both glycerol and ethylene glycol (solvents) around 1800 KOH·g^−1^ [41] increases the overall hydroxyl number of the obtained polyol, as mentioned before [42]. The low hydroxyl number at 1:3 may indicate that limited solvent availability and high viscosity hinder effective depolymerization and promote condensation reactions, resulting in lower hydroxyl density. The substantial rise at 1:10 suggests that an excess of solvent is required to stabilize reactive intermediates and prevent recondensation, allowing the preservation of hydroxyl-rich fragments. Therefore, a high material-to-solvent ratio not only improves liquefaction yield but also increases the hydroxyl functionality of the resulting polyol.

### 3.3. Thermogravimetric Analysis

TGA and DSC thermograms are widely used to understand the thermal behavior of a sample for a specific temperature interval. The TGA technique allows us to know the mass loss of the analyzed sample as temperature increases at a specific rate. On the other hand, the DSC technique shows the amount of heat exchanged between the equipment and the sample as compared with a reference. These two techniques are generally combined to associate each mass loss with a specific thermal transition or a specific reaction. For the case of the DSC technique, a negative value of the measured heat flow is associated with an exothermic transformation in which heat is released from the sample to the outer medium. Contrarily, a positive heat flux refers to an endothermic transformation.

**Figure 4:**
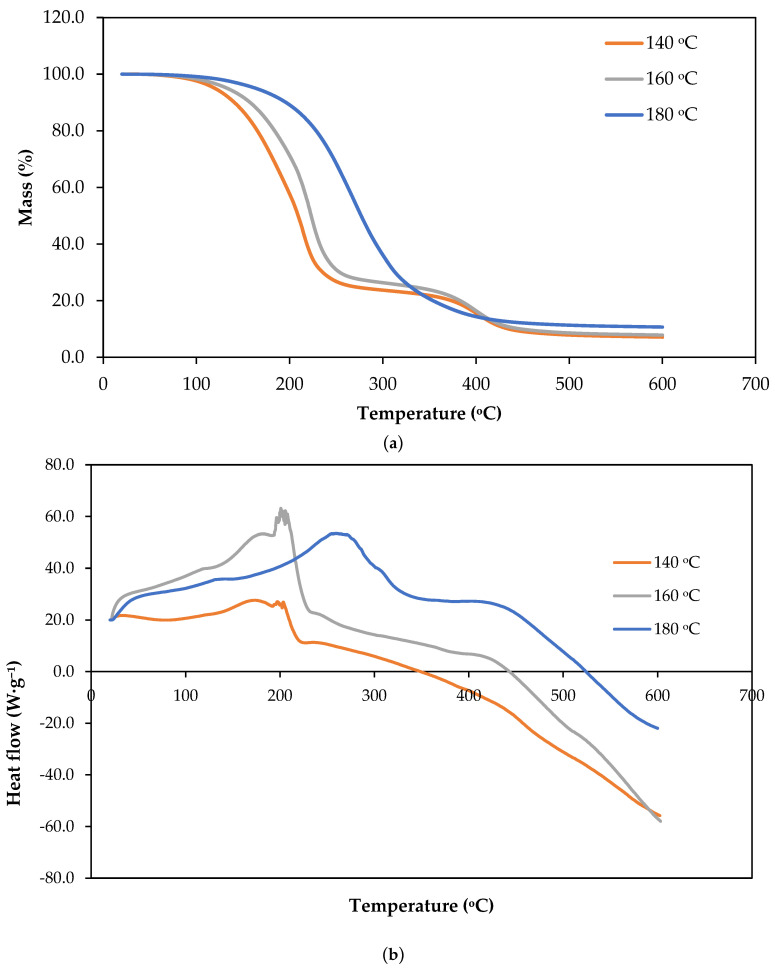
(**a**) Thermogravimetric analysis (TGA) curves of polyols obtained from the liquefaction of *Ruscus* at 140 °C, 160 °C, and 180 °C. The onset degradation temperatures (T_0_) were approximately 99 °C, 110 °C, and 130 °C, respectively, while the maximum degradation temperatures (Tmax) were approximately 202 °C, 205 °C, and 270 °C, respectively. (**b**) Differential Scanning Calorimetry (DSC) curves of the corresponding polyols.

Figure 4 shows the thermogravimetric analysis (TGA) curves of polyols obtained from the liquefaction of *Ruscus* at 140 °C, 160 °C, and 180 °C, expressed as the remaining mass (%) as a function of temperature. Polyols obtained at 140 °C and 160 °C exhibit a multi-step thermal degradation profile, which is typical of polyols derived from lignocellulosic biomass. The detailed analysis of these curves shows an initial plateau in which very minimal mass loss (ca. 1–2%) occurs for the temperature interval between 20 °C and 100 °C, indicating extremely dry or low-volatile biopolyols, which was expected since all the polyols were completely dried before analysis.

The polyols exhibit two clear inflection points, indicating a two-step thermal degradation process. The first inflection, occurring below approximately 200–220 °C, corresponds to the loss of low-molecular-weight compounds such as residual solvents, moisture, and light polyol fractions generated during liquefaction. This step is more pronounced for the 140 °C sample, suggesting a higher proportion of volatile or weakly bound components due to less extensive depolymerization during liquefaction. The decomposition between 150 °C and 310 °C has been attributed to the degradation of glycerol [43,44] that has a boiling point of 290 °C, and therefore evaporates at this temperature. The second inflection, observed between roughly 250 and 400 °C, is associated with the decomposition of heavier liquefaction products, including oligomeric carbohydrate- and lignin-derived structures. Similar results were presented before with the TGA of *Eucalyptus pellita* wood polyol showing a significant degradation in the range of 305–474 °C, which was attributed to the decomposition of cellulose and lignin [44,45]. After this region, the mass decreases more slowly and approaches a plateau, with final residues of about 7–10% at 600 °C, indicating limited char formation.

In contrast, the polyol produced at 180 °C shows a single broader mass loss inflection line, extending from approximately 200 to 350 °C. This behavior suggests that the degradation processes observed as two distinct steps in the lower-temperature samples have merged into one dominant decomposition event. The absence of a clearly separated low-temperature step indicates a reduced fraction of volatile and low-molecular-weight compounds, likely due to further reactions occurring during liquefaction at higher temperatures. Shi et al. [46] studied cellulose liquefaction in polyhydric alcohols and compared the TGA of cellulose and cellulose/glycerol and concluded that the degradation of glycerol occurred at lower temperatures (250 °C) than cellulose (around 300 °C), which confirms that the polyol obtained at 180 °C has a smaller amount of low-molecular-weight compounds.

The broader degradation region and the smoother mass decrease point to a more homogeneous material with a narrower distribution of thermally labile components. The higher residual mass at 600 °C, around 10–12%, suggests increased formation of thermally stable, condensed structures. This confirms that the liquefaction temperature plays a critical role in controlling both the thermal behavior and structural complexity of *Ruscus*-derived polyols.

The TGA curves of these polyols (Figure 4a) exhibit a multistep thermal degradation behavior consistent with previously reported TG/DTG results for liquefied rapeseed cake and glycols [47]. The main weight-loss regions observed at 180–270 °C and 270–420 °C are attributed to the decomposition of glycol components and liquefied lignocellulosic fragments derived from hemicellulose, cellulose, and lignin, respectively. Increasing liquefaction temperature shifts the degradation to higher temperatures and increases char residue, indicating improved thermal stability of the resulting polyols.

The thermal behavior of the bio-polyols was characterized via Differential Scanning Calorimetry (DSC) (Figure 4b) using a convention where positive heat flux denotes endothermic transitions. The thermograms illustrate that the polyols undergo significant endothermic deviations, likely associated with the volatilization of residual components or the thermal cleavage of bonds formed during the liquefaction process. A clear trend is observed: increasing the liquefaction temperature from 140 °C to 180 °C shifts the onset of these endothermic events toward higher temperatures, indicating enhanced thermal stability in the resulting products.

The polyol liquefied at 140 °C exhibited a sharp endothermic peak at approximately 200 °C. This suggests that the material contains a high concentration of low-boiling-point intermediates or thermally unstable fragments, such as hemicellulose-derived monomers and unreacted liquefaction agents, which require relatively low energy to undergo phase transition or decomposition. For example, this temperature correlates closely with the atmospheric boiling point of ethylene glycol, 197.6 °C, suggesting that at this lower liquefaction temperature, a significant portion of the ethylene glycol remains as unreacted or weakly associated free solvent. The presence of this sharp peak indicates that 140 °C is insufficient to achieve high conversion or to chemically incorporate the solvent into the lignin/cellulose matrix through stable glycosidic or ether linkages. These results are supported by the TGA analysis done before.

Similarly, the sample liquefied at 160 °C showed a broader, complex endothermic profile centered near 215 °C, representing an intermediate stage of biomass conversion where the most labile fractions have been partially stabilized or removed.

The bio-polyol synthesized at 180 °C demonstrated the highest thermal resistance, with the primary endothermic event delayed until approximately 260 °C. The significant shift and the broader, flatter nature of this peak suggest that the higher liquefaction temperature promoted more extensive depolymerization and subsequent stabilization of the biomass constituents. This suggests that at 180 °C, the polyol reaches a more homogenous and chemically robust state, likely due to the more complete conversion of the crystalline cellulose regions and the formation of stable glycosidic derivatives. Consequently, the 180 °C liquefaction temperature is optimal for producing bio-polyols with superior thermal endurance, which is a critical requirement for their subsequent application in high-performance polymer synthesis, such as polyurethane foams.

### 3.4. Temperature-Dependent Viscosity

Polyol viscosity is a critical parameter for polyurethane processing because it strongly influences mixing behavior, bubble nucleation, cell growth, and drainage during the early stages of foam formation. Consequently, variations in polyol viscosity can significantly affect foam morphology, density, and mechanical performance.

**Figure 5:**
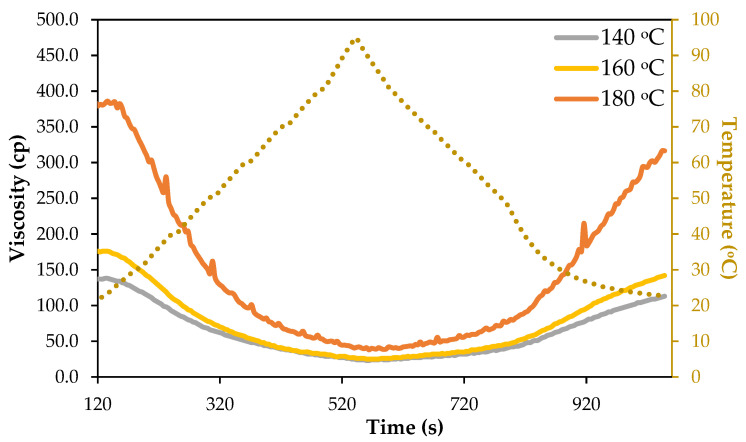
Temperature-dependent apparent viscosity profiles of bio-polyols obtained from the liquefaction of *Ruscus aculeatus* branches at 140, 160, and 180 °C for 60 min, measured using a Rapid Visco Analyzer (RVA 4500).

Figure 5 illustrates the temperature-dependent viscosity profiles of polyols obtained by liquefaction of *Ruscus* branches at 140 °C, 160 °C, and 180 °C for 60 min. Viscosity values are expressed as apparent viscosity (cP) and were recorded under controlled thermo-mechanical conditions. The profiles should be interpreted as comparative rheological behavior rather than absolute viscosity measurements, as the RVA operates under instrument-specific shear conditions.

All polyols were fully dried prior to rheological measurements, ensuring that the observed viscosity differences arise from intrinsic structural features rather than residual moisture effects.

For all samples, viscosity decreases during the heating phase and increases upon cooling, reflecting the typical inverse relationship between viscosity and temperature due to enhanced molecular mobility and reduced intermolecular interactions at elevated temperatures. However, pronounced differences are observed between polyols obtained at different liquefaction temperatures, highlighting the strong influence of liquefaction severity on polyol structure and rheological behavior.

Despite this common thermal behavior, significant differences are observed among polyols produced at different liquefaction temperatures. The polyol obtained at 180 °C exhibits markedly higher viscosity throughout the entire time interval (reaching 385 cp), whereas those produced at 140 °C and 160 °C display substantially lower viscosities (137 cp, 176 cp, respectively) with similar overall profiles. These observations are further supported by the RVA-derived rheological parameters summarized in Table 1. The increase in peak viscosity and final viscosity with liquefaction temperature indicates the formation of more complex molecular structures and stronger intermolecular interactions. Additionally, the higher breakdown and setback values observed for the 180 °C polyol suggest lower structural stability under thermo-mechanical conditions and a greater tendency for molecular reassociation during cooling.

Similar results were presented before for bark-based polyols synthesized via solvent liquefaction using a polyethylene glycol/glycerol cosolvent system at reaction temperatures of 90, 130, and 160 °C [48]. The values were much higher than the ones obtained here (maximum 385 cp), which is probably due to the higher viscosity of PEG compared to ethylene glycol. These differences indicate that liquefaction temperature has a pronounced effect on the chemical structure and molecular organization of the resulting polyols. Notably, this occurs even though the hydroxyl number decreases with increasing liquefaction temperature, from 1038 mg KOH·g^−1^ at 140 °C to 932 mg KOH·g^−1^ at 160 °C and 916 mg KOH·g^−1^ at 180 °C, unlike the increase observed before from 228 to 331 KOH·g^−1^ [48].

During the cooling phase, viscosity increases more sharply for the polyol obtained at 180 °C than for the other samples, and the viscosity–temperature path does not retrace that observed during heating. This hysteresis behavior indicates irreversible structural modifications occurring during liquefaction, such as chain growth, oligomer aggregation, and enhanced hydrogen bonding. These irreversible changes are less pronounced for the polyols obtained at 140 °C and 160 °C, consistent with their lower liquefaction severity and reduced extent of secondary reactions. Overall, the results demonstrate that liquefaction temperature plays a critical role in controlling the balance between hydroxyl functionality and molecular architecture in *Ruscus*-derived polyols. Lower liquefaction temperatures favor the formation of low-viscosity, highly functionalized polyols, whereas higher temperatures promote condensation reactions that increase viscosity while reducing hydroxyl number. These structural and rheological differences are expected to strongly influence the reactivity, processability, and performance of the polyols in subsequent applications such as polyurethane synthesis.

The viscosity profile of the polyol obtained at 180 °C is more irregular and has several peaks. This can be due to lignin fragments generated by bond cleavage that can undergo rapid condensation and re-polymerization reactions, leading to the formation of highly aromatic, rigid structures with limited solubility in the glycerol/ethylene glycol medium. Upon cooling, or even during temperature fluctuations under shear, these condensed structures may partially precipitate or exist as dispersed solid particles or gel-like domains within the liquid polyol. The presence of such solid or semi-solid fractions can strongly affect rheological measurements. As the measurement proceeds, these particles may temporarily form networks or agglomerates, increasing resistance to flow and causing abrupt increases in apparent viscosity. Under continued shear or changing temperature, these agglomerates can break apart or redistribute, leading to sudden decreases in viscosity. This dynamic formation and disruption of particle networks would manifest as irregular viscosity profiles with multiple peaks, as observed for the 180 °C polyol.

The polyol produced at 140 °C is expected to result in foams with coarser and less uniform cells, due to the relatively low initial viscosity, which permits rapid bubble expansion and reduces viscoelastic resistance during early foaming [17]. While this polyol would be easy to handle during mixing, the resultant foam may exhibit lower mechanical performance and dimensional stability. On the other hand, the polyol obtained at 180 °C, with a viscosity of 390 cP, would likely generate foams with very fine and uniform cellular structures and improved dimensional stability, consistent with literature observations that higher-viscosity polyols enhance control over bubble growth and suppress coalescence [17,18]. However, the high viscosity may pose challenges during processing, potentially requiring more vigorous mixing or elevated temperatures to achieve homogenous foam formation [19,21]. Similar values were reported for the polyols obtained by liquefaction of Kraft lignin with a mixture of PEG 400/Glycerol catalyzed with different catalysts with viscosity ranging from 118–346 cp [34]. Likewise, the liquefaction of some agricultural residues, rice, oilseed rape, wheat, and corn stover, with a mixed solvent system of PEG400 and ethylene glycol with a sulfuric acid catalyst, has shown that viscosity varied from the 215 cp of wheat straw to 102 cp of rice straw polyol [17].

The viscosity of these polyols is, however, lower than the viscosity of polyols from southern pine obtained with a mixture of PEG 400/glycerol that presented a viscosity ranging from 1420 to 1720 cp [33] or from cryptomeria between 1733 and 4340 cp [49]. In accordance with Kong et al. [50] in industrial applications, polyols are primarily valued for their high hydroxyl numbers and ease of processing due to low viscosity which they considered to be between 100–10,000 cp.

From an industrial perspective, the scalability of biomass liquefaction processes depends on factors such as solvent consumption, energy demand, and process integration. The high liquefaction yields obtained in this study within relatively short reaction times suggest favorable conditions for potential scale-up. The use of glycerol and ethylene glycol mixtures is advantageous in this context because these solvents are relatively inexpensive. Glycerol is a secondary product of biodiesel production, and ethylene glycol can be produced from bioethanol.

### 3.5. FTIR-ATR

**Figure 6:**
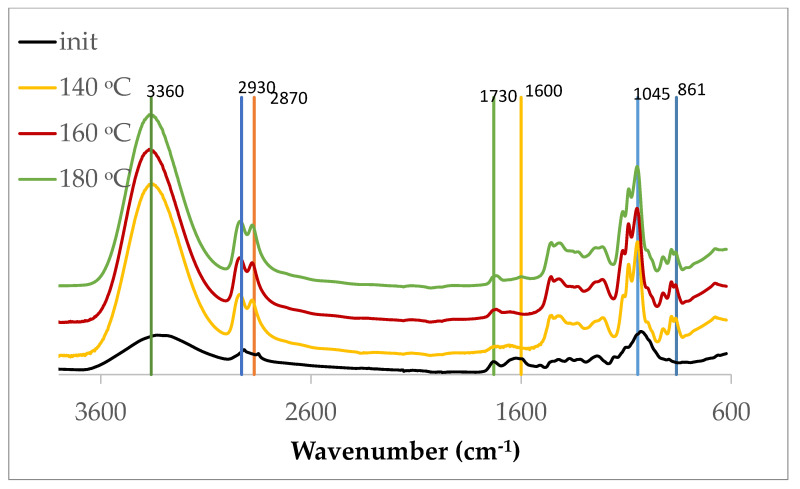
FTIR-ATR spectra of the initial biomass and polyols obtained from liquefaction of *Ruscus aculeatus* branches at 140, 160, and 180 °C for 60 min.

The FTIR-ATR spectra of the polyols produced from *Ruscus* branches at different liquefaction temperatures show the typical absorption bands of lignocellulosic-derived polyols, with noticeable variations in intensity as a function of temperature (Figure 6). These variations indicate progressive chemical transformations during liquefaction.

All liquefied materials display a broader band centered at ca. 3360 cm^−1^, assigned to the O–H stretching vibrations, significantly stronger than that of the initial material, reflecting the formation of hydroxyl-containing compounds during depolymerization. Nevertheless, it is known that IR light typically penetrates deeper into liquids than into solids due to their lower refractive index and density, which leads to more pronounced absorption bands in liquid samples. This band is observed in all samples but shows a progressive intensity decrease with increasing liquefaction temperature. This trend indicates a reduction in free hydroxyl groups at higher temperatures, which is consistent with the experimentally observed decrease in OH index. The reduction in hydroxyl functionality can be associated with condensation reactions, etherification, and possible dehydration processes occurring under more severe liquefaction conditions.

The absorption bands around 2930 and 2870 cm^−1^ correspond, respectively, to antisymmetric and symmetric stretching vibrations of the aliphatic –CH_2_ and –CH_3_ groups. These signals indicate the presence of aliphatic chains originating from degraded polysaccharides and lignin side chains. Their persistence across all samples suggests that the aliphatic backbone of the liquefied biomass is preserved, while small intensity differences may reflect temperature-dependent fragmentation and rearrangement reactions. The main difference in these signals is observed in relation to the starting material. The symmetric stretching in the initial material shows its maximum at 2850 cm^−1,^ while in the liquefied samples it shifts to 2870 cm^−1^ with an increase in intensity, becoming greater than the signal at 2930 cm^−1^. A similar behavior was observed before for liquefied olive branches [25].

A weak but discernible band around 1730 cm^−1^ is assigned to C=O stretching vibrations of ester and/or carbonyl groups. The presence of this band in the liquefied material indicates oxidation and/or esterification reactions between degradation products and the liquefaction solvent. The increased definition of this band, at higher temperatures, points to greater ether bond breakage and the formation of carbonyl-containing compounds as liquefaction conditions become stronger. This increase with liquefaction temperature has been reported previously [48].

The signal observed near 1600 cm^−1^ is associated with aromatic C=C stretching vibrations, mainly derived from lignin structures that almost disappear during liquefaction.

The strong absorption centered at around 1045 cm^−1^, with shoulders at ca. 1085 cm^−1^ and 1100 cm^−1^, are assigned to C–O and C–C stretching vibrations of alcohols, ethers, and polysaccharide-derived structures. This complex of signals is particularly important for polyols, as it reflects the abundance of hydroxyl-bearing carbon–oxygen bonds. There are no significant variations in its intensity for all temperatures studied.

The absorption band observed near 860 cm^−1^ can be assigned to C–H deformation and ring vibration modes associated with pyranose structures [51] originating from carbohydrate-derived components of the liquefied wood polyol, but can also be related to aromatic C–H out-of-plane bending vibrations [52], confirming the presence of lignin-derived aromatic structures in polyols.

Although no new functional groups were identified, variations in band intensities provide evidence of structural modifications during liquefaction. In particular, the decrease in O–H intensity and the enhancement of carbonyl-related bands support the occurrence of dehydration, condensation, and oxidation reactions. These spectral changes, although subtle, are consistent with the trends observed in hydroxyl number and thermal behavior, supporting the proposed reaction mechanisms.

However, FTIR analysis mainly provides qualitative information, and therefore these observations should be interpreted as indicative trends rather than definitive structural evidence.

The FTIR spectra for the polyols obtained at 180 °C at different times (15, 30, and 60 min) do not show significant differences (Supplementary Figure S1).

The development of bio-based polyols from lignocellulosic biomass contributes to the broader field of sustainable polymer research by enabling the partial substitution of petroleum-derived polyols in polyurethane formulations. Future research should explore strategies such as blending biomass-derived polyols with conventional or low-viscosity polyols in order to tailor rheological properties and optimize foam processing conditions.

The results obtained in this work are consistent with previously reported studies on the liquefaction of lignocellulosic biomass using polyhydric alcohol solvents. Several studies have demonstrated that reaction temperature, solvent composition, and biomass particle size are key parameters controlling biomass depolymerization and polyol formation. The high liquefaction yields and favorable rheological properties observed for *Ruscus aculeatus* polyols highlight the potential of this biomass as a promising renewable feedstock for the production of bio-based polyols and polyurethane materials

## 4. Conclusions

This study demonstrates that *Ruscus aculeatus* L. branches are a promising lignocellulosic feedstock for the production of bio-polyols via liquefaction, achieving high conversion yields comparable to or exceeding those reported for other biomasses. Among the parameters investigated, temperature was identified as the dominant factor, with liquefaction yields exceeding 90% at 180 °C, while reaction time showed a comparatively minor influence, indicating potential for reduced energy consumption.

Particle size and solvent ratio significantly affected process efficiency, with smaller particles and higher solvent proportions enhancing mass transfer, conversion, and hydroxyl functionality. The decrease in hydroxyl number with increasing temperature reflects the progression of dehydration and condensation reactions under more severe conditions. Thermal and rheological analyses revealed that higher liquefaction temperatures promote the formation of more homogeneous, thermally stable, and higher-viscosity polyols, consistent with increased molecular weight and secondary reactions. These changes directly influence processability and final material performance. The obtained polyols exhibit distinct property profiles depending on liquefaction temperature: low-temperature polyols (140 °C) are characterized by high hydroxyl numbers and low viscosity, making them suitable for flexible polyurethane systems, while high-temperature polyols (180 °C) show higher viscosity and thermal stability, favoring rigid or high-density applications. Intermediate conditions (160 °C) provide a balance between reactivity and stability.

Overall, the results highlight the critical role of liquefaction conditions in tailoring polyol properties for specific applications. The findings confirm the strong potential of *Ruscus aculeatus* L. as a sustainable raw material for bio-based polyols, while also emphasizing the importance of process optimization to balance reactivity, stability, and processability in industrial applications.

## Figures and Tables

**Table 1:** RVA-derived rheological parameters of bio-polyols obtained at different liquefaction temperatures.

Rheological Properties					
Polyol	Peak Time (s)	Peak Viscosity (cP)	Breakdown Viscosity (cP)	Final Viscosity (cP)	Setback Viscosity (cP)
180 °C	260	385.00	350.00	314.00	279.00
160 °C	200	176.00	151.00	142.00	117.00
140 °C	220	137. 00	115.00	113.00	90.00

## Data Availability

All data generated or analyzed during this study are included in this published article [and its Supplementary Information files], and raw data is available from the corresponding author on reasonable request.

## Supplementary Materials

The following supporting information can be downloaded at: https://www.mdpi.com/article/10.3390/polym18070880/s1. Figure S1. FTIR-ATR spectra of the initial material and polyols obtained from the liquefaction for 15, 30 and 60 min at 180 °C
