# Supplementary material for: Liquefaction of Ruscus aculeatus Branches into Bio-Polyols: Process Optimization and Polyol Characterization
Source: Polymers (Basel). 2026 Apr 3;18(7):880. doi: 10.3390/polym18070880 (PMC13074745; doi:10.3390/polym18070880)
Supplement: Supplementary file 1 [file polymers-18-00880-s001.zip › polymers-4204453-supplementary.pdf]

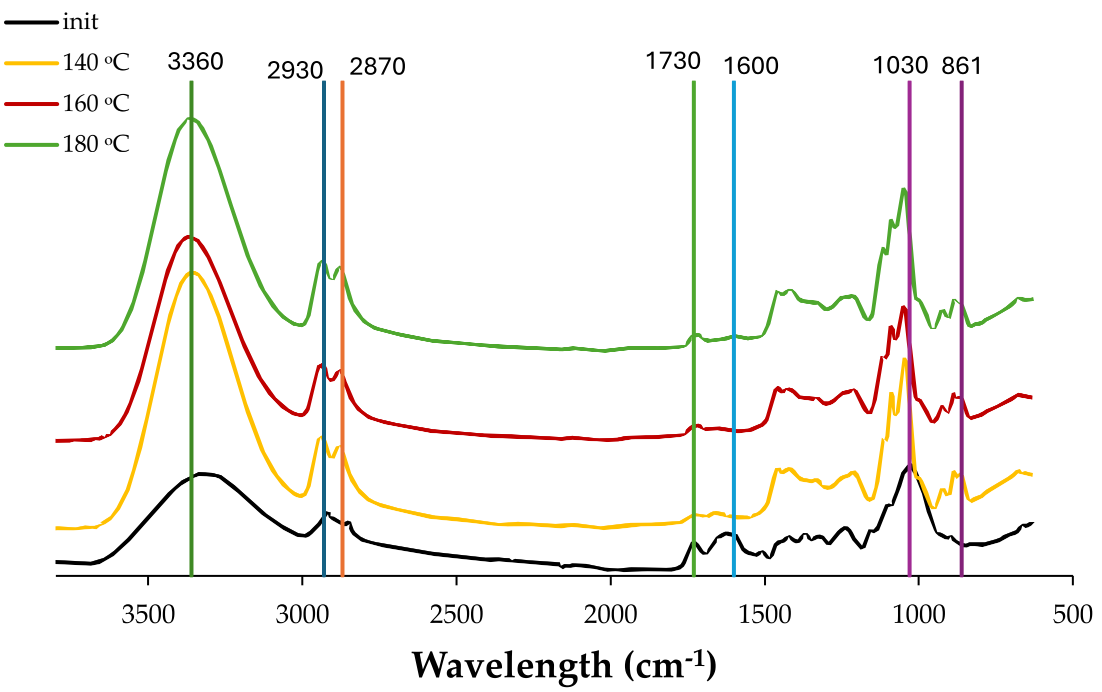

Figure S1. FTIR-ATR spectra of the initial material and polyols obtained from the liquefaction for 15, 30 and 60 min at 180 °C.
